# Mazabraud’s Syndrome

**DOI:** 10.5334/jbsr.2698

**Published:** 2022-07-29

**Authors:** Masset Adrien, Bottosso Nadège, Kurth William

**Affiliations:** 1 Chu liège, BE

**Keywords:** Mazabraud, Syndrome, fibrous dysplasia, intramuscular myxoma

## Abstract

**Teaching point:** Mazabraud’s Syndrome defines the association of bone fibrous dysplasia and intramuscular myxoma, sometimes with pathological fractures or deformities in the lower limbs.

## Case Report

**Figure 1:**
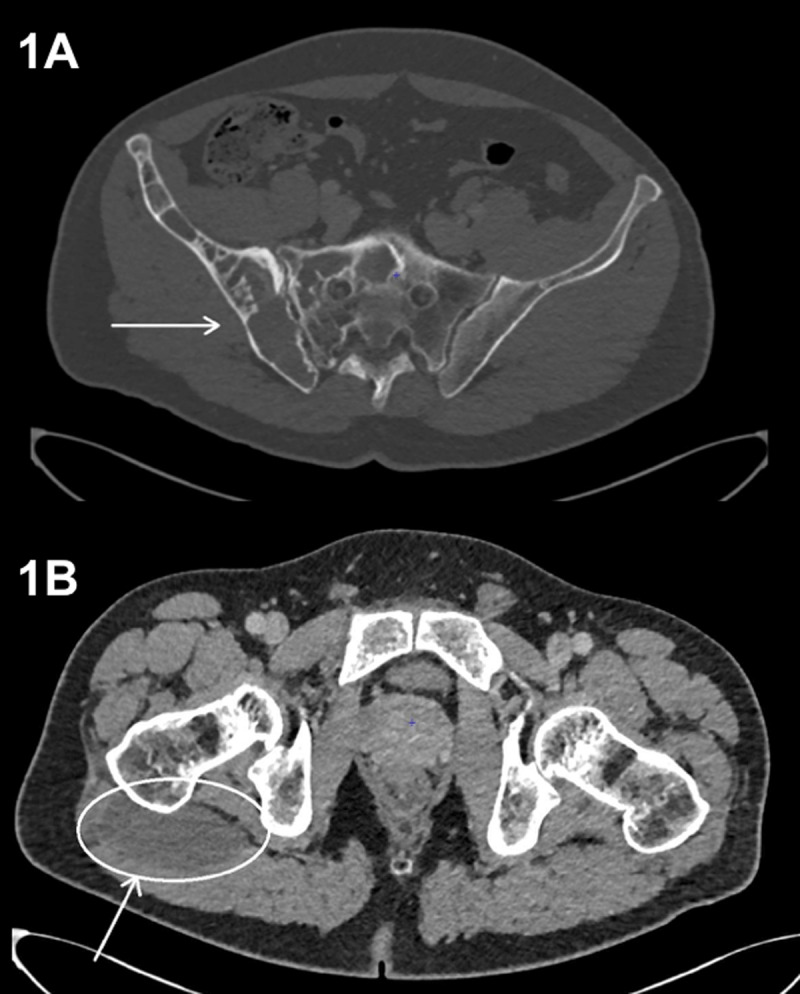


A 44-year-old man presented to the emergency room with left abdominal pain which required abdominal computed tomography (CT), evidencing left ureteral lithiasis, multiple osteolytic lesions with sclerotic margins on lower ribs, thoracolumbar spine, pelvis and right femur (Figure 1A, arrow). A large ovoid intramuscular gluteus maximus hypodensity was also found, suggesting a collection (Figure 1B, circle).

As the patient indicated intermittent right gluteal and lumbar pain over the last months without any functional impairment, pelvic magnetic resonance imaging (MRI) was performed for characterization.

**Figure 2:**
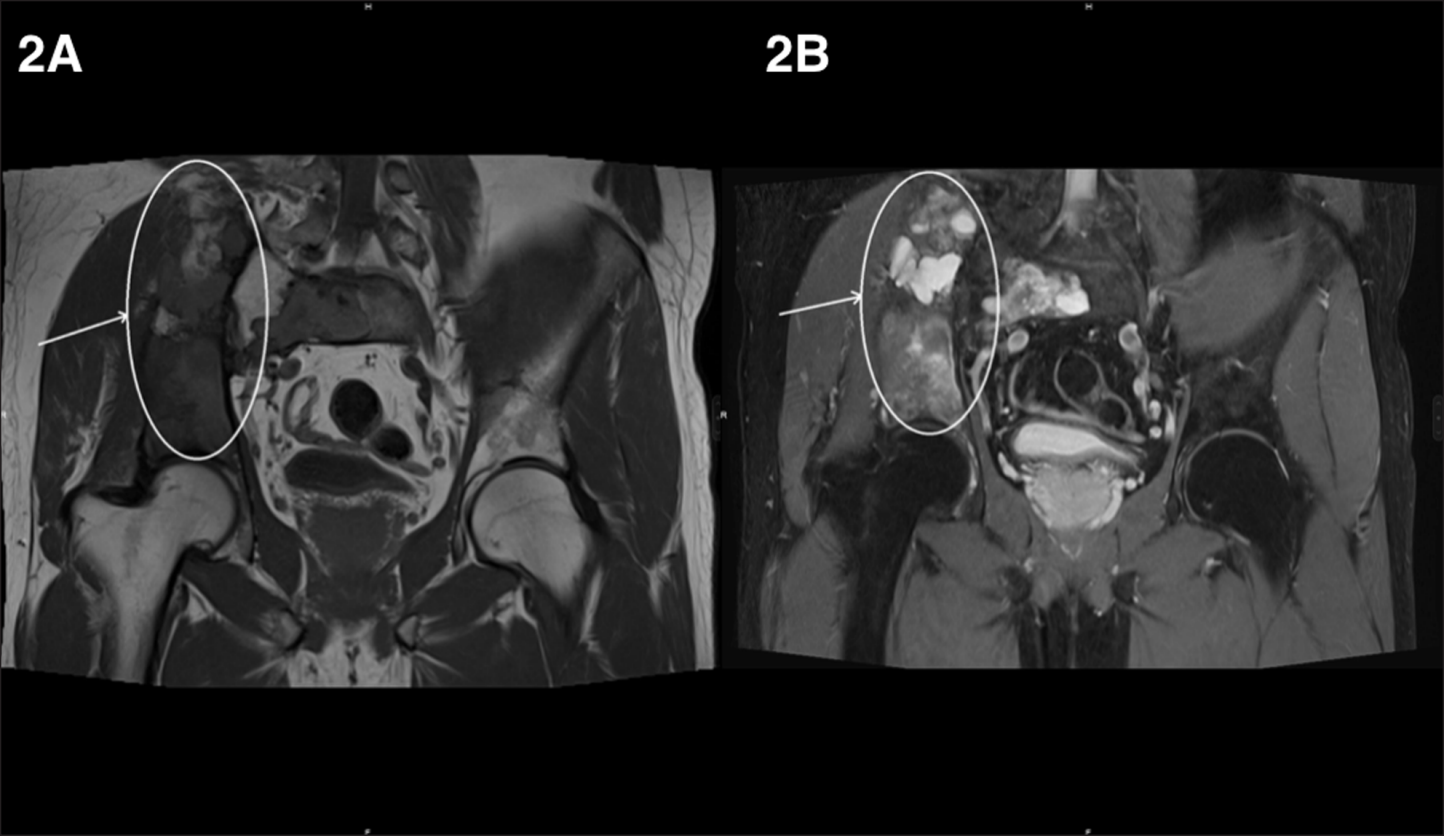


It showed numerous intra-osseous lesions with hypointense and heterogenous hypersignal respectively on T1-weighted (Figure 2A, circle) and T2-weighted (Figure 2B, circle) images. Enhancement was heterogeneous on post-Gadolinium sequences.

**Figure 3:**
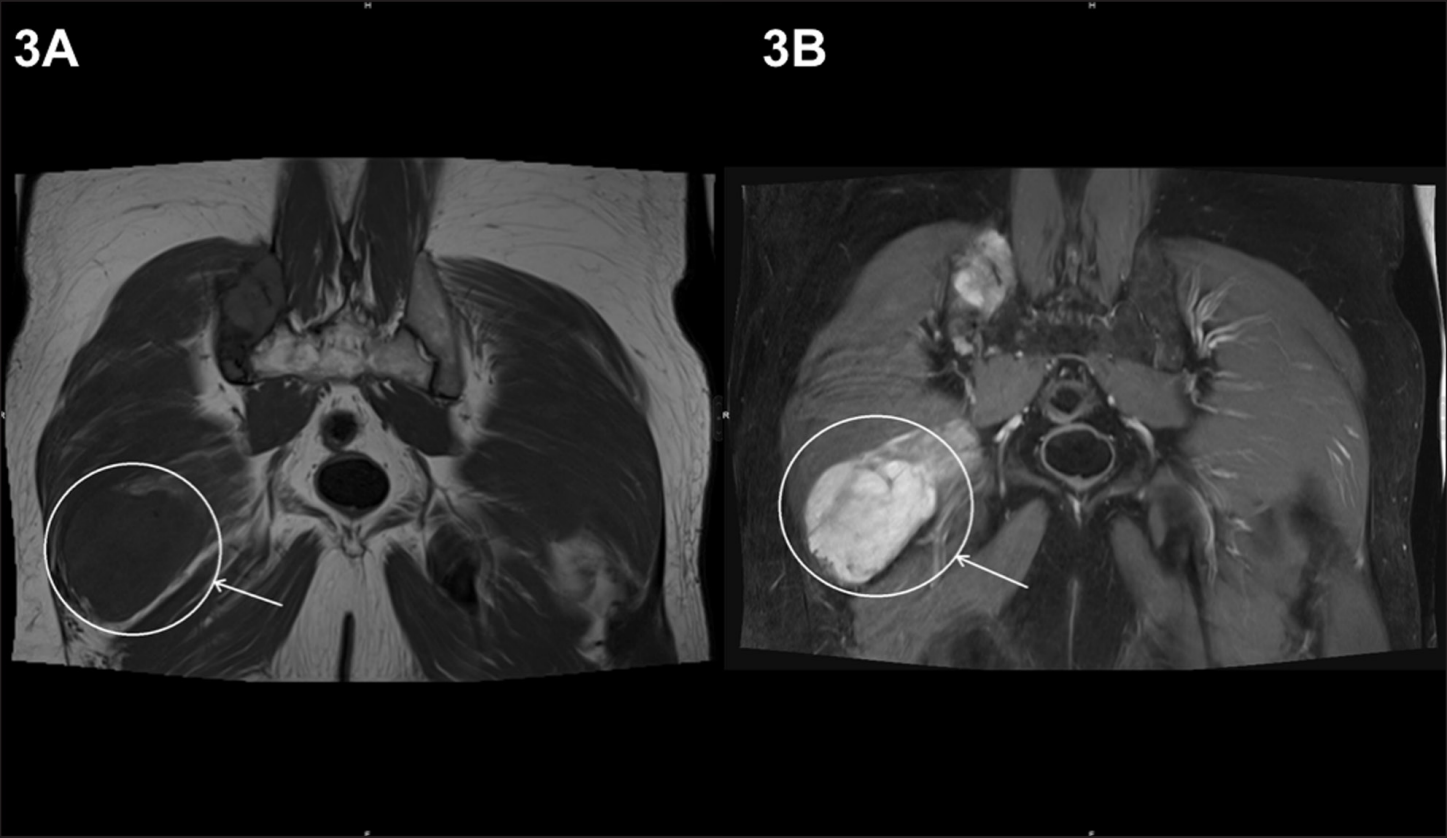


The intramuscular lesion was T1 hypointense and T2 hyperintense (Figure 3A and 3B, respectively, circles).

The diagnosis of fibrous dysplasia and intramuscular myxoma was retained also known as Mazabraud’s syndrome. Routine follow-up was started with a six-month MRI.

## Comment

Mazabraud’s syndrome defines the association of bone fibrous dysplasia and intramuscular myxoma [1]. The prevalence is approximately 1/1,000,000 and 2 of 3 are women [1]. It generally onsets in the fifth decade; 40 years for fibrous dysplasia and 47 years for myxoma [1]. Fibrous dysplasia is defined as the transformation of normal bone and bone marrow towards an abnormal fibrous tissue [1]. The lesions are generally polyostotic (77%) with pelvis and femur as first localisations. Malignant evolution is extremely rare but can justify the long-term follow-up with fibrous dysplasia aggressive form [1].

Intramuscular myxoma are benign mesenchymal neoplasms consisting of undifferentiated spindle cells, myxoid stroma and collagen fibres. Multiples myxomas in the quadriceps (or generally the lower limbs) are the most frequent situation [1].

The pathology is caused by inactivating mutations of the GNAS1 gene that induce aberrant cell proliferation at different stages of cellular maturation. Because the physiopathology involves early-stage cells, Mazabraud’s syndrome can present wide clinical forms such as bone pain, deformities or pathological fractures, but patients are sometimes asymptomatic or paucisymptomatic like ours [1].

On radiographs, fibrous dysplasia looks like a radiolucent lesion, appearing in “ground-glass” matrix, with sclerotic bone rim. The bone cortical stays intact [1].

MRI is the gold standard exam to evaluate Mazabraud’s syndrome, for both diagnosis and follow-up with detection of malignant evolution [1].

Fibrous dysplasia appears generally with hyposignal on T1-weighted images and hypersignal on T2 sequence surrounding by hypointense on both T1 and T2 sequences border. There is generally low heterogeneous contrast enhancement. Intramuscular Myxoma shows low intensity on T1 and hyper intensity on T2 [1].

Treatment is variable, from clinical and imaging follow-up to orthopedic surgery for symptomatic patient. Long-term bone antiresorptive agents (bisphosphonates) like zoledronate would be an alternative. It seems reduce the volume of myxoma but doesn’t have any effect on dysplasia [1].
